# Hormonal regulation of thirst in the amphibious ray-finned fish suggests the requirement for terrestrialization during evolution

**DOI:** 10.1038/s41598-019-52870-7

**Published:** 2019-11-08

**Authors:** Yukitoshi Katayama, Yoshio Takei, Makoto Kusakabe, Tatsuya Sakamoto

**Affiliations:** 1 Laboratory of Physiology, Atmosphere and Ocean Research Institute, The University of Tokyo, 5-1-5 Kashiwanoha, Kashiwa, Chiba 277-8564 Japan; 2 Faculty of Science, Toho University, 2-2-1 Miyama, Funabashi, Chiba 274-8510 Japan; 3 Faculty of Science, Shizuoka University, 836 Otani, Suruga, Shizuoka, Shizuoka 422-8529 Japan; 4 Ushimado Marine Institute, Faculty of Science, Okayama University, 130-17 Kashino, Setouchi, Okayama 701-4303 Japan

**Keywords:** Evolution, Animal behaviour

## Abstract

Thirst has evolved for vertebrate terrestrial adaptation. We previously showed that buccal drying induced a series of drinking behaviours (migration to water–taking water into the mouth–swallowing) in the amphibious mudskipper goby, thereby discovering thirst in ray-finned fish. However, roles of dipsogenic/antidipsogenic hormones, which act on the thirst center in terrestrial tetrapods, have remained unclear in the mudskipper thirst. Here we examined the hormonal effects on the mudskipper drinking behaviours, particularly the antagonistic interaction between angiotensin II (AngII) and atrial natriuretic peptide (ANP) which is important for thirst regulation in mammalian ‘forebrain’. Expectedly, intracerebroventricular injection of ANP in mudskippers reduced AngII-increased drinking rate. ANP also suppressed the neural activity at the ‘hindbrain’ region for the swallowing reflex, and the maintenance of buccopharyngeal water due to the swallowing inhibition may attenuate the motivation to move to water. Thus, the hormonal molecules involved in drinking regulation, as well as the influence of buccopharyngeal water, appear to be conserved in distantly related species to solve osmoregulatory problems, whereas hormonal control of thirst at the forebrain might have been acquired only in tetrapod lineage during evolution.

## Introduction

Drinking behaviour is important for body fluid regulation throughout the vertebrates. In mammals, the sensory circumventricular organs (sCVOs) in the forebrain, which lack the blood-brain barrier, are critical for motivation (i.e., thirst) for a series of drinking behaviours. The subfornical organ (SFO), one of the sCVOs, has recently been found in mice to receive information about the local sensation of buccopharyngeal water^1^, as well as signals from blood factors^2,3^ such as osmolarity, angiotensin II (AngII), and atrial natriuretic peptide (ANP). Intracerebroventricular (ICV) injection of ANP attenuated the AngII-induced thirst by acting on the SFO^4,5^. The neuro/endocrine regulatory mechanism of drinking behaviour is poorly understood in non-mammalian vertebrates. Thirst as a regulator of drinking in amphibians has been suggested^6^. AngII induces thirst and ‘cutaneous drinking’ (absorption through the ventral skin) in toads^7^. In ray-finned fishes, water is always available so only imbibition is needed, and no searching for water is required^8^. Indeed, the above systemic hormones may regulate swallowing through the area postrema (AP) in the hindbrain^9–11^, and no thirst center in the forebrain has yet been found in ray-finned fishes.

The land invasion by vertebrates in the evolutionary process occurred not only in lobe-finned fishes that led to tetrapods but also in ray-finned fishes, independently of the tetrapod lineage^12,13^. For instance, mudskipper fishes originated from the marine environment^14,15^ but spend a significant portion of their time out of water, as a means of predator avoidance and also to forage thus they have evolved numerous physiological and behavioural traits associated with an amphibious lifestyle^14,16^. Unlike amphibians and fresh-water fishes, the mudskipper skin secretes Cl^−^ as with the surface area of the gills which is most of the body-surface area of general seawater fish and the transcutaneous water uptake has not been described^17–20^. Our very recent study of the mudskipper, *Periophthalmus modestus*^21^, has shown that AngII acts centrally at the area postrema (AP) to induce swallowing of buccal water as in other fishes. The loss of buccal water thereby induced the migration to water for refilling, which was the first evidence of thirst in ray-finned fish. This thirst perceived through local sensation (i.e., ‘local thirst’) appears to be primarily important for a series of drinking behaviours in the mudskipper, suggesting a common requirement of thirst for terrestrialization throughout the vertebrates^21^. Local sensation of buccal drying may relay to a possible thirst center, but the neural bases in the forebrain remain unclear even in mammals^22^. The mudskipper might be a unique experimental model to investigate evolutionary process of thirst and hormonal influences. In particular, antidipsogenic factor(s) can allow mudskippers to retain buccal water which is important for their physiological and behavioural traits in terrestrial lifestyles (e.g., opercular/branchial respiration, ammonia secretion, terrestrial foraging)^15,23^. Analyses of possible antidipsogenic functions of ANP in mudskipper thirst regulation are highly intriguing in order to know how hormonal control contributed to vertebrate terrestrialization, compared with the known mechanisms in tetrapods.

To clarify the hormonal regulation of the mudskipper thirst, we here analysed whether and how ICV injections of AngII and ANP affect drinking behaviour. We found that ANP attenuated an AngII-increased drinking rate and the neural activity at the AP. We revealed the importance of the AP as the site of action of these hormones, unlike in mammals. Despite such antagonistic effects of ANP on AngII, ICV injection of ANP alone prolonged the period of time in water. Since this might be an intrinsic action of another natriuretic peptide secreted from the heart, B-type natriuretic peptide (BNP) which shares a receptor with ANP^24–26^, we also examined the role of BNP. Finally, we discuss the hormonal basis of the requirements for the transition of vertebrates from aquatic to terrestrial habitats during evolution.

## Results

### Identification of ANP and BNP in the mudskipper heart

**Figure 1:**
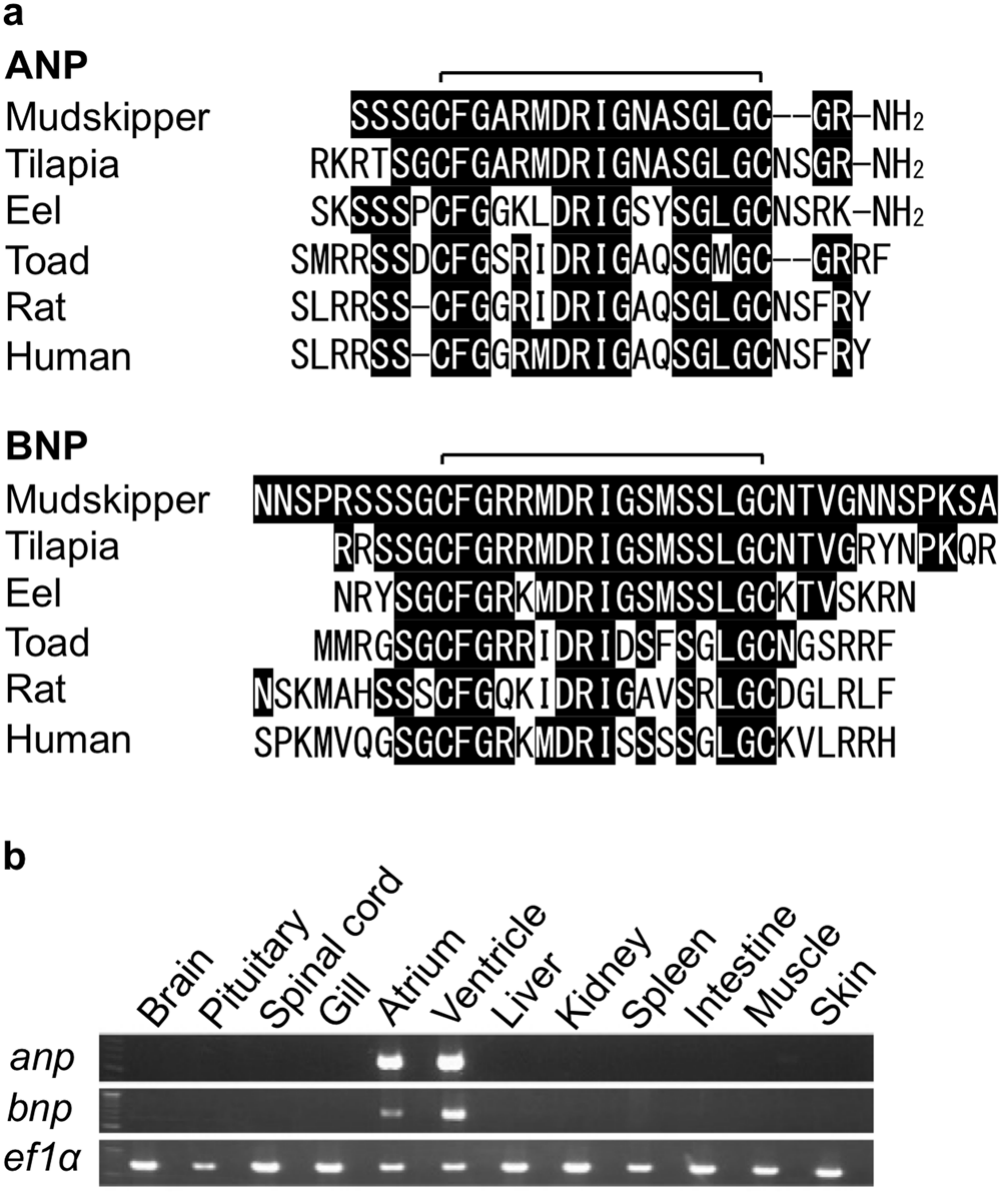
Identification of atrial natriuretic peptide (ANP) and B-type natriuretic peptide (BNP) in the mudskipper. (**a**) The amino-acid sequences of the mature forms of ANP and BNP deduced from cloned cDNA sequences. Highlighted residues are identical to those in the mudskipper sequences. Disulfide bonds are indicated by brackets. (**b**) Tissue distribution of *anp* and *bnp* mRNAs. Elongation Factor 1 α (*ef1α*) mRNA was used as a control for each cDNA sample. Reverse transcription control without template was not detected in any tissue. The gel pictures were cropped and their full-length gels are shown in Supplementary Fig. 2.

cDNAs of *anp* and *bnp* were cloned from the mudskipper heart (GenBank Accession numbers LC348996 for *anp*, and LC348997 for *bnp*). Molecular phylogenetic analyses of the cDNA sequences are shown in Supplementary Fig. 1. Mudskipper *anp* and *bnp* are typical of ray-finned fish *anp*s and *bnp*s, respectively. Mature sequences of the amino acids deduced from the cDNA sequences (Fig. 1a) are substantially conserved among the corresponding orthologs. The peptides of mudskipper ANP and BNP were synthesized based on these sequences and used for following physiological studies. The tissue distribution of the mRNAs of *anp* and *bnp* was also examined in the mudskipper. Both were detected only in the atrium and ventricle (Fig. 1b) similarly to *anps* and *bnps* of other fish species^27,28^.

### Antagonistic effect of ANP in drinking induced by AngII

**Figure 2:**
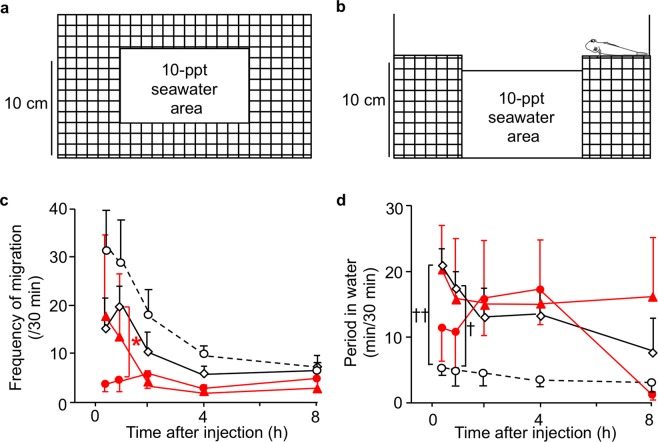
Amphibious behaviour in mudskippers after intracerebroventricular (ICV) injection of ANP in the presence of angiotensin II (AngII). (**a**,**b**) Schematic diagram of experimental tank used to observe amphibious behaviour of mudskippers. Top (**a**) and side (**b**) views. 10-ppt seawater is close to their natural environment and almost identical to the salinity of body fluids, and thus was chosen for the experiments. (**c,d**) Timecourse changes in amphibious behaviour. The frequency of migration (**c**) and the period of time in water (**d**) were measured after ICV injection with 0.1 μl/g of 3 × 10^−8^ M AngII alone (*n* = 6, open diamonds), 3 × 10^−8^ M (*n* = 4, red triangles) to 3 × 10^−7^ M ANP (*n* = 7, red circles) in the presence of AngII, and vehicle (*n* = 8, open circles). The parameters were measured at 0.5 h (0.25–0.75 h), 1 h (0.75–1.25 h), 2 h (1.75–2.25 h), 4 h (3.75–4.25 h) and 8 h (7.75–8.25 h) after injections. No apparent sex differences were found in these effects. Three-way ANOVA (with time course, hormones, and doses) and Tukey’s post-hoc test were used for statistical analysis. Data are shown as means ± standard error of the mean (s.e.m.). ^*^p = 0.012, ^†^*p* = 0.009, ^††^p = 0.0022.

**Figure 3:**
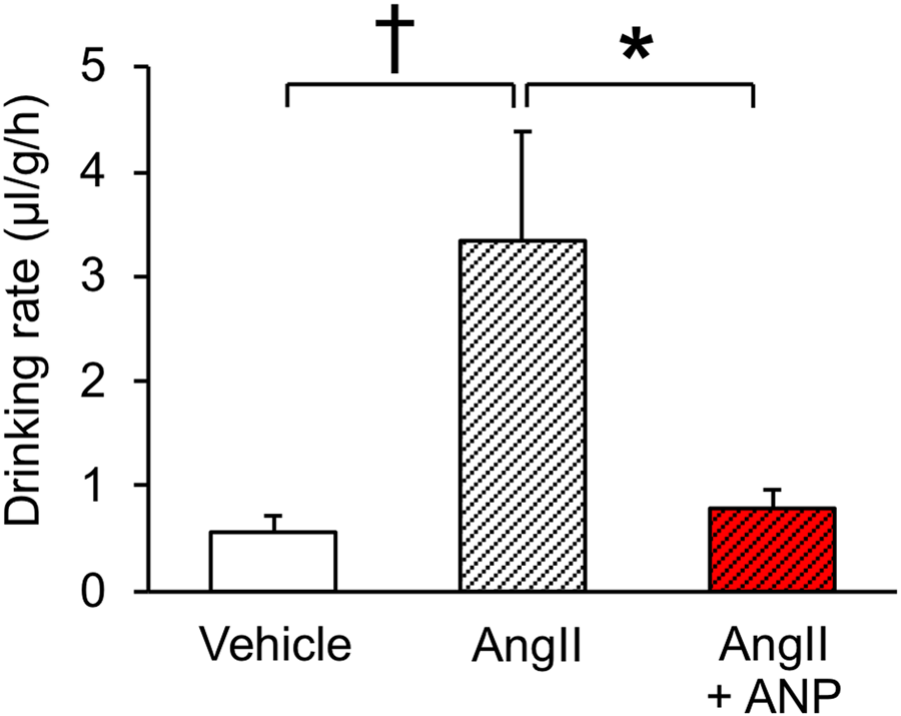
Water intake in mudskippers after ICV injection of ANP in the presence of AngII. Fish were put in a columnar tank containing phenol red in 10-ppt seawater for an hour and ingested water was measured after ICV injection with 0.1 μl/g of 3 × 10^−8^ M AngII alone (*n* = 7), 3 × 10^−7^ M ANP in the presence of AngII (*n* = 7), and vehicle (*n* = 7, handling control). No apparent sex differences were found in these effects. Kruskal-Wallis test and Steel-Dwass post-hoc test were used for statistical analysis. Data are shown as means ± s.e.m. ^*^p = 0.048, ^†^*p* = 0.017.

The antagonistic interaction between AngII and ANP, which was demonstrated in mammalian thirst^4,29^, was first examined in the thirst-motivated behaviours of mudskippers. The ICV administration of AngII increased period of time in water as in our previous study^21^. Concurrent ICV treatment with ANP and with AngII decreased the ‘frequency of migration’ after 30 min compared with the AngII treatment alone (Fig. 2c), although this combined treatment did not significantly affect the period in water (Fig. 2d). The amount of ingested water was increased by AngII, and ANP abolished this effect (Fig. 3). On the other hand, little phenol-red absorption by the skin (i.e., the transcutaneous/transbranchial water uptake) was observed. These results indicate that ANP attenuates oral-drinking induced by AngII.

### The hindbrain AP as the site of the antagonistic action of ANP

**Figure 4:**
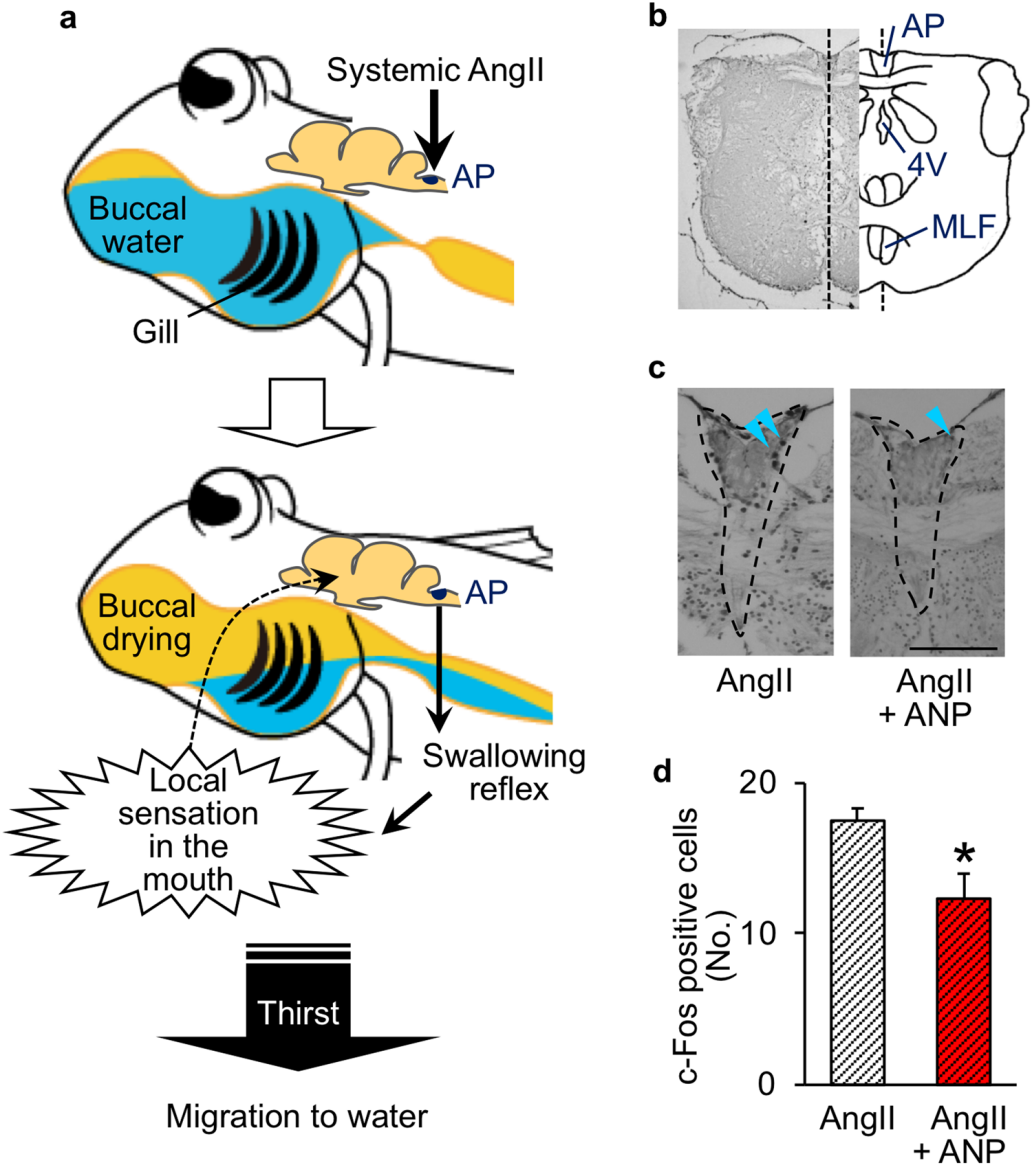
The antagonistic interaction between ANP and AngII in the mudskipper brain. (**a**) The regulation of a series of drinking behaviours by AngII in the mudskipper. Localization of the area postrema (AP) in the hindbrain (i.e., the reflex center) is shown. AngII acts primarily on the AP to induce swallowing of buccal water, and loss of buccal water evokes thirst by the local sensation^21^. (**b**) A photomicrograph showing the c-Fos immunoreactivity in a cross section of the mudskipper AP, and a schematic of the section. Broken lines indicate the midline. 4 V, fourth ventricle. MLF, medial longitudinal fasciculus. (**c**) The c-Fos positive cells in the AP after ICV injection with AngII alone or ANP in the presence of AngII. Broken lines delineate the AP, and arrowheads indicate the representative positive cells. Scale bar: 100 μm. (**d**) Number of c-Fos positive cells in the AP after the injections. Data are shown as means ± s.e.m. of 5 fish. *p = 0.014 with unpaired *t*-test.

Direct antagonistic interaction between AngII and ANP was well described in the mammalian SFO^4,29^. In ray-finned fishes, the SFO has not been identified, but the hindbrain AP, which regulates swallowing, is known to be the sole site of AngII action^9,21^. In our previous study of the mudskipper^21^, the enhanced neural activity was limited to the AP neurons which expressed angiotensin type 1 receptor like gene. This data indicated that AngII acted primarily on the hindbrain to induce buccal drying and subsequent drinking behaviours (Fig. 4a). Therefore, we examined the effect of ANP on the immunoreactivity of c-Fos, a marker of neuronal activity, at the AP. Concurrent ICV injection of ANP with AngII attenuated the increase in the number of c-Fos immunopositive neurons at the AP by the treatment of AngII alone^21^ (Fig. 4c,d), but not in the medial longitudinal fasciculus where the neurons were not considered to be involved in drinking^30^ (see Supplementary Results). These results indicate antagonistic interaction between AngII and ANP at the AP neurons.

### Distinct effects of ANP alone on migration and water intake

**Figure 5:**
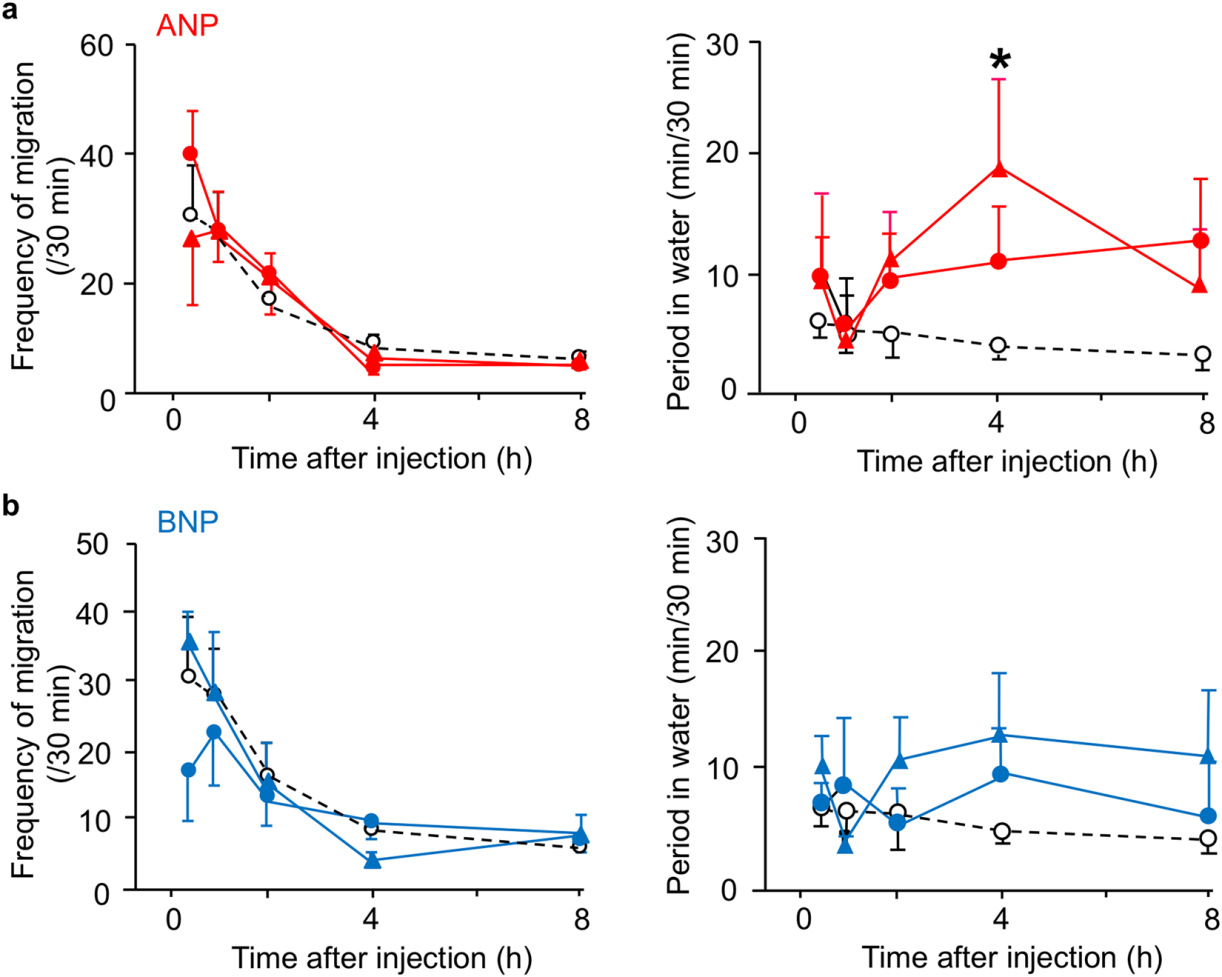
Amphibious behaviour in mudskippers after ICV injection of ANP (**a**) or BNP (**b**) alone. The frequency of migration and period in water were measured after injections with 0.1 μl/g of 3 × 10^−6^ M (*n* = 5–6, solid circles), 3 × 10^−8^ M (*n* = 4–6, solid triangles), and vehicle (*n* = 8, control, open circles) of ANP or BNP. No apparent sex differences were found in these effects. Two-way ANOVA and Tukey’s post-hoc test were used for statistical analysis. Data are shown as means ± s.e.m. *p = 0.012 versus controls.

**Figure 6:**
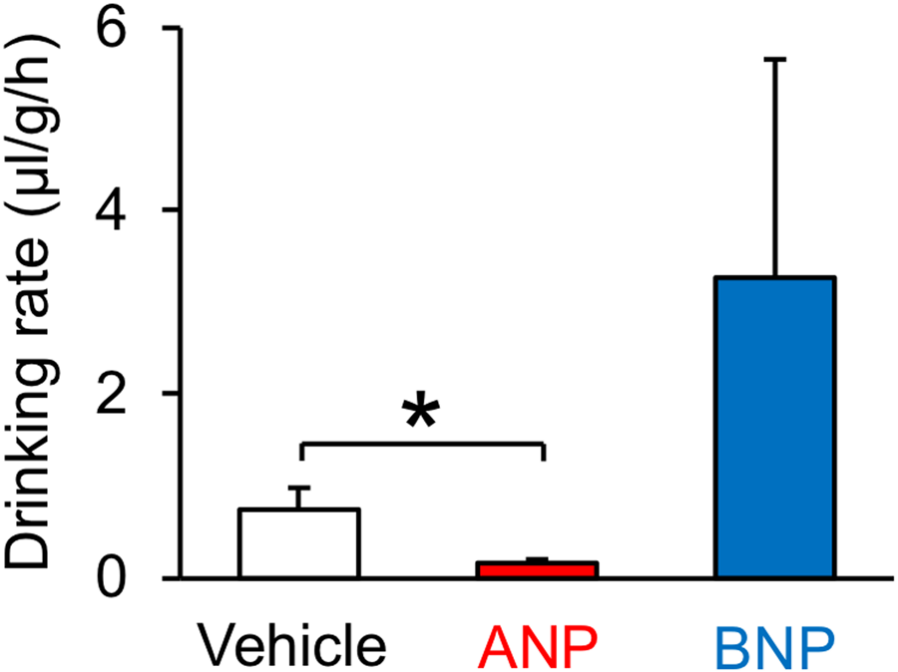
Water intake in mudskippers after ICV injection of ANP or BNP alone. Drinking rate was measured after injection with 0.1 μl/g of 3 × 10^−6^ M ANP (*n* = 5), BNP (*n* = 5), or vehicle (*n* = 8). No apparent sex differences were found in these effects. Kruskal-Wallis and Steel post-hoc test were used for statistical analysis. Data are shown as means ± s.e.m. *p = 0.025.

In addition, the effect of ANP alone on a series of drinking behaviours was examined in the mudskipper, since relevant functions of ANP except for the antagonism with AngII are known in several vertebrates^26,31^. For two hours after the ICV injection of ANP, no significant change in the migration into water was shown (Fig. 5a). At four hours after the injection, however, ANP prolonged the period of time in water. Because this effect of ANP is not consistent with the above antagonism and may potentially reflect an intrinsic action of BNP, another cardiac NP, we also examined the effect of BNP. There were no significant differences between control and BNP-injected groups in the period of time in water or in the frequency of migration (Fig. 5b). When we specifically examined water intake, ANP alone inhibited water intake and BNP did not show such effect (Fig. 6).

## Discussion

**Figure 7:**
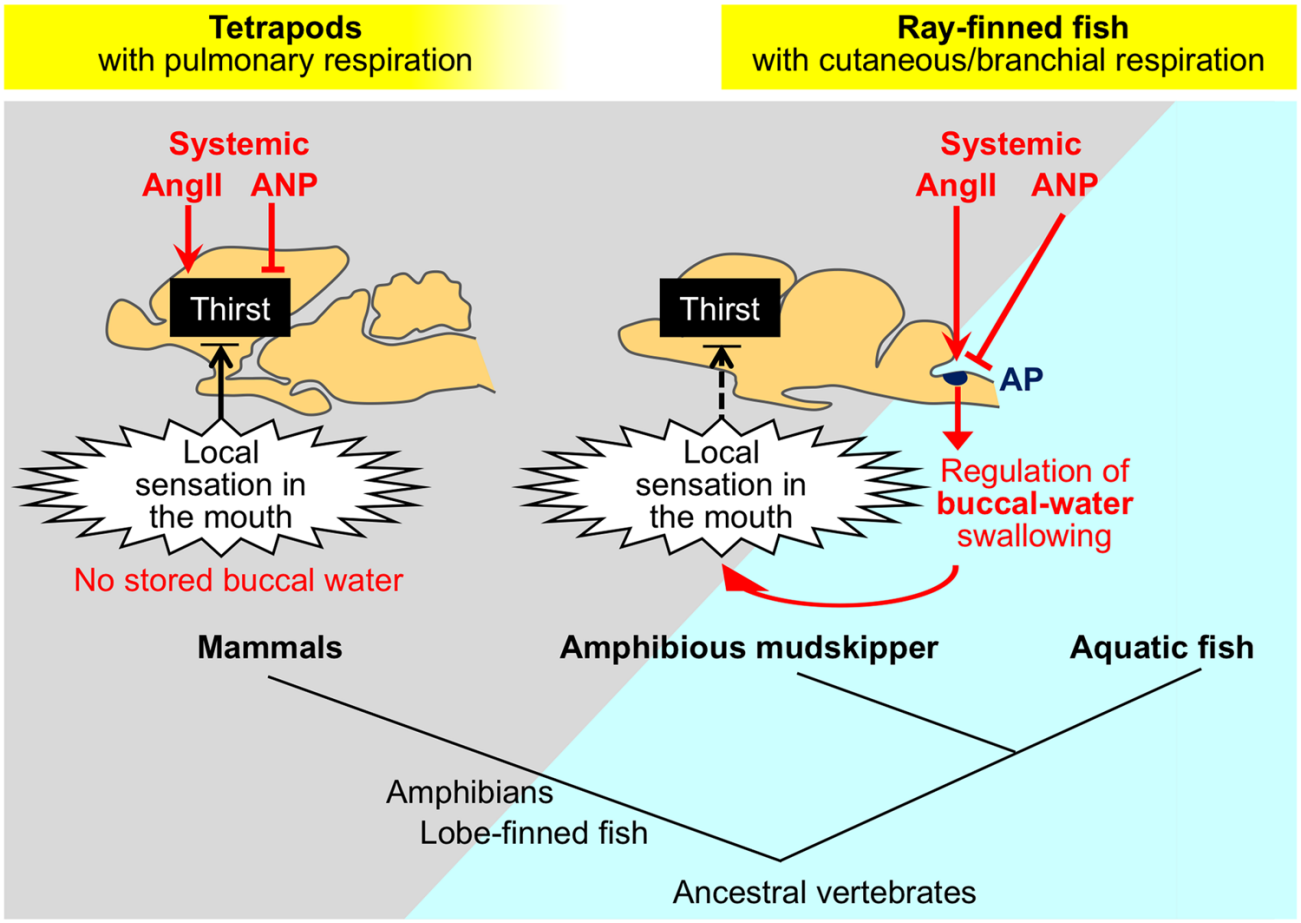
Evolution of the thirst regulation by AngII and ANP as well as by local sensation in the mouth. Regulation of drinking by AngII and ANP appears to have been acquired both in tetrapod and ray-finned fish lineages. Furthermore, thirst induced by local sensation in the mouth seems to have been acquired both in tetrapods and the amphibious ray-finned fish (mudskipper) for invasion onto the land during the evolution^21^. Although the important hormonal molecules appear to be conserved through vertebrates, the neural bases of these hormonal actions may differentiate between the ray-finned fish and tetrapod lineages according to their lifestyles. Tetrapods do not store water in the buccal cavity, and must move for oral/cutaneous drinking via the motivation signal (i.e., thirst). The direct actions of the hormones and the local sensation (i.e., dry mouth) are integrated in the forebrain (i.e., the center of emotional behaviour) to regulate the motivation. On the other hand, the mudskipper (*Periophthalmus modestus*) can swallow water without movement to water, because water is stored or present in the mouth of these fish. Thus, the primary actions of AngII and ANP can be limited to their hindbrain APs (i.e., the reflex center). When the mudskipper is on land, loss of buccal water by swallowing induces local thirst to evoke migration to water probably through the forebrain. In contrast to the tetrapod forebrain, a possible thirst center in the mudskipper brain appears to be independent from the major site of hormonal action in the hindbrain. It is assumed that ancestral vertebrates (agnathan, cartilaginous fish, and marine lobe-finned fish) whose body fluids are isosmotic have not developed the regulation of drinking.

Here we showed for the first time that AngII and ANP act antagonistically on the drinking behaviour of amphibious mudskipper as in tetrapods. In tetrapods, these hormones primarily act on the forebrain to regulate thirst^10,29^. In the mudskipper, however, our results showed the antagonistic interaction in the hindbrain AP that regulates swallowing, and the subsequent sensation of buccal water may control the thirst (i.e., the motivation to move to water)^21^. Our comprehensive analyses using the mudskipper, which is independent of the tetrapod lineage^12,13^, suggest the importance of the evolutionarily-conserved thirst regulation by local sensation (i.e., local thirst) rather than by direct actions of hormones at the forebrains. Although the hormonal molecules important for drinking appear to be conserved through vertebrates, the neural bases of their actions may differ between the ray-finned fish and tetrapod lineages (Fig. 7).

Since *anp* mRNA was detected only in the mudskipper heart, the sCVOs that lack a blood-brain barrier^11,32,33^ appear to be the direct central target site of systemic ANP secreted from the heart, as considered in other vertebrates^29,34,35^. The hindbrain CVO (the AP) in ray-finned fishes has been considered to be the target site of ANP as well as that of AngII^8,9,11^. Further work on identification and localization of ANP receptor in the mudskipper brain is required. Our amphibious mudskipper (*Periophthalmus modestus*) as well as aquatic fishes can swallow water without movement to water and the primary site of hormonal action can be limited to the hindbrain, because water is present in the mouth of these fish; some mudskippers store water in the buccal cavity even when they are on land^21,23,36^. In contrast, tetrapods and some other mudskipper species including *Periophthalmus barbarus* and *Periophthalmodon schlosseri*^37,38^ do not store water in the buccal cavity, and thus must move to water for drinking (taking water into the mouth–swallowing). In tetrapods, complex neural-circuitry convergence of the direct hormonal actions and buccal-water local sensation in the SFO^1,22,39^ appears to be required for thirst regulation (Fig. 7). Since the major site of hormonal actions in the mudskipper hindbrain seems to be independent from the potential center of local thirst regulation^18^, this local-thirst center can be specifically analyzed by excluding the neural circuit from hormonal input. Thus, this fish may be used as the excellent and unique model.

Regulation of drinking by AngII and ANP, which is herein shown in the mudskipper, appears to have been acquired independently both in tetrapod and ray-finned fish lineages during the terrestrial/hyperosmotic adaptation. It is assumed that agnathan, cartilaginous fish and possibly ancestral vertebrates, whose body fluids are isosmotic to environmental seawater, have not developed such regulation of drinking^40,41^, whereas regulation of blood pressure by these hormones is well conserved throughout the vertebrates^26,42^. Because some mudskippers store water in the buccal cavity on land^21,23^, the inhibition of swallowing by ANP should promote this buccal-water storage on land and lead to satiation of local thirst (Figs 4a and 7). This ANP function may allow the mudskipper to stay on land longer^14,15^ and to feed on land with its hydrodynamic tongue^23^. Such hormonal contributions to local thirst regulation might have evolutionarily occurred also in other ray-finned fish taxa that invaded land^12,13^. Some amphibious fishes that do not store water in the buccal cavity^37,38^ cannot drink water by swallowing on land, and they might have a mechanism similar to that of tetrapods.

The antagonistic action of ANP on AngII in migration into water was only transient and was not convincingly evident, which may relate to the opposite significant preference for an aquatic habitat at 4 hours after the injection of ANP alone (Fig. 5a). It is possible that the antidipsogenic effect is masked by the secondary effect due to increased cortisol secretion stimulated by ANP, possibly through the pituitary-interrenal axis^43^, since cortisol prolongs the period spent in water^44^. We also speculated that secondary effects of ANP may reflect an intrinsic action of BNP which shares a receptor with ANP. However, there were no significant effects of BNP injections on a series of drinking behaviours of mudskippers in the present study, unlike in other vertebrates including eels^24–26^. Because there were no significant changes in the mRNA levels of *anp* or *bnp* after mudskippers moved to terrestrial conditions (Supplementary Table 1), NPs may play a somewhat minor role in the migration to water. On the other hand, BNP is also involved in the transduction of itch sensation in the mammalian spinal cord^45^. Therefore, mudskippers might sense drying of the skin on land, and then, through NP action, seek to moisten the skin^46^. It will be intriguing to examine whether injection of NPs specifically changes the behaviour for moistening. NPs might have acquired various central actions for terrestrial adaptation in addition to regulation of oral drinking.

In summary, the antagonistic interaction between AngII and ANP for swallowing also occurs in the amphibious mudskipper, suggesting the universal importance of these regulatory hormones in drinking behaviour. In the fish, however, unlike in tetrapods, these direct actions were found only in the neural pathway for reflex swallowing to influence the storage of buccal water. Together with our recent study^21^, local thirst sensation, which is similar to ‘dry mouth’ or ‘anticipatory thirst’ in mammals analysed only recently^1,47^, is proposed in the mudskipper as a primarily important motivation to move to water^48^. Such regulation of thirst by these sensory and hormonal inputs may have evolved in distantly related species^21^ in order to solve osmoregulatory problems, although the hormonal role has appeared to differentiate according to the modes of respiration. Understanding the emergence of these thirst mechanisms should provide a fresh insight into the acquired systems for the terrestrialization of vertebrates.

## Materials and Methods

### Animals

One year-old mudskippers (*P. modestus*) of both sexes weighing 3 to 5 g were collected from the estuary of the Fujii River, which flows into the Inland Sea of Seto (34°N: 134°E). As previously described^44,49,50^, plasma ions, differentiation of osmoregulatory organs, hormonal status, and amphibious behaviours in mudskippers under varying conditions have been examined. Since we previously showed that no sex differences were found in their amphibious behaviour, both sexes of fish were used^21^. Fish were acclimated for 2–5 weeks in laboratory tanks (3 L). These fish–collected from brackish water–were held in the tanks that contain diluted seawater (10 ppt, 149 mM Na^+^, 176 mM Cl^−^, 3.8 mM Ca^2+^, 346 mOsml/kg), which is almost isotonic to mudskipper plasma. Small plates were placed in each tank to allow mudskippers an opportunity to climb onto them. All specimens were maintained at room temperature of 22–25 °C under a daily photoperiod cycle of 12-h light/12-h dark (lights on at 7:00 a.m.) and were fed daily with Tetrafin flakes (TetraWerke, Melle, Germany). Fish were anesthetized in 0.01% tricaine methanesulfonate (Sigma, Tokyo, Japan) neutralized with sodium bicarbonate before handling. All experiments were approved by the Animal Experiment Committee of the University of Tokyo and Okayama University, and performed in accordance with the manuals prepared by the committees.

### cDNA cloning of mudskipper *anp* and *bnp*

The procedures for *anp* and *bnp* cDNA cloning were modified from the previous study^35^. After anesthesia, the hearts were isolated from mudskippers and immediately frozen in liquid nitrogen. Total RNA was extracted using ISOGEN (Nippongene, Toyama, Japan). Single-stranded cDNA was prepared from 1 μg of heart RNA using High Capacity cDNA Reverse Transcription kit (Thermo Fisher Scientific, MA, USA). Partial sequences of *anp* and *bnp* were amplified by PCR with degenerate primers: forward primer 5′-AGYYGBHTVCWGGAYCTSCT-3′, reverse primer 5′-CTSGMRKTYCCDATBCGRTCCA-3′ for *anp* and forward primer 5′-TCMGRGAGYTYCTBTCAKC-3′, reverse primer 5′-CCVAYDGTGTTRCAVCCMAGAGA-3′ for *bnp*, respectively. cDNAs were amplified with high-fidelity Ex Taq DNA polymerase (TaKaRa, Tokyo, Japan), ligated into pGEM T-easy plasmid (Promega, Madison, WI, USA). Amplified products were sequenced by an ABI3130*xl* DNA sequencer (Applied Biosystem, Foster City, CA, USA) and BigDye Terminator v3.1 Cycle Sequencing Kit (Applied Biosystems, Foster City, CA, USA). Based on the determined partial sequences, gene-specific primers: forward primer 5′-CTGCTCAGTGCGCGCCGCTCCTCCTC-3′, reverse primer 5′-CCTCTGCCACAGCCCAGGCCACTAGCG-3′ for *anp* and forward primer 5′-CCCAGAACCTGCGGACTGTCCGGAA-3′, reverse primer 5′-GAGCTCATGGAGCCGATGCGATCCA-3′ for *bnp* were designed and used for 3′ and 5′ RACE. Finally, cDNAs encompassing the whole coding regions were amplified using gene-specific primers designed based on the sequences obtained by the RACE method: 5′-ATGAGGGCCGCATTCGTGTGGG-3′, 5′-AGACCAACCTGCAAAATGCGC-3′ for *anp* and, 5′-ATGGGGAGAGTAGGATCAGAATTGG-3′, 5′-CAAATGTAAACCTTTAATGAACAAAAC-3′ for *bnp*. Deduced amino acid sequences of ANP and BNP were compared with those of other vertebrates and a phylogenetic tree was generated by maximum likelihood method using the MEGA6 program at http://www.megasoftware.net/. GenBank accession numbers of genes and mRNAs used in this analysis are NM_006172.3 (human ANP), NM_002521.2 (human BNP), NM_012612.2 (rat ANP), NM_031545.1 (rat BNP), AF429999.3 (cane toad ANP), DQ113654.1 (toad BNP), LC348996 (mudskipper ANP), LC348997 (mudskipper BNP), AB087283.1 (Mozambique tilapia ANP), AB087284.1 (tilapia BNP), AB019372.1 (Japanese eel ANP), and AB179820.1 (eel BNP).

### Tissue distribution of *anp* and *bnp* mRNAs

Tissue distribution of *anp* and *bnp* mRNAs was examined by RT-PCR. The brain, pituitary, spinal cord, gill, atrium, ventricle, liver, kidney, spleen, intestine, muscle, and skin were sampled from mudskippers and quickly frozen in liquid nitrogen. Total RNA was extracted and cDNAs of tissues were prepared from 1 μg of total RNA as mentioned above. Elongation Factor 1 α (*ef1α*) cDNA was used for an internal standard. Gene-specific primers for *anp* and *bnp*, which amplify cDNA of the whole coding regions, were designed as described above. Gene-specific primers for *ef1α* were 5′-GAGCGTGAGCGTGGTATCACCAT-3′ and 5′-GTCTGCCTCATGTCACGCAC-3′. The PCR conditions were: 94 °C, 2 min followed by 30 cycles (*ef1α*) or 35 cycles (*anp* and *bnp*) of denaturation (94 °C, 30 s), annealing (59 °C for *ef1α*, 64 °C for *anp* and *bnp*, 30 s), and extension (72 °C, 5 min). The amplified DNA fragments were electrophoresed on 1% agarose gels and detected by ethidium bromide staining.

### ICV injection

Mudskipper AngII (NRVYVHPF), ANP (SSSGCFGARMDRIGNASGLGCGR-NH_2_), and BNP (NNSPRSSSGCFGRRMDRIGSMSSLGCNTVGNNSPKSA) were synthesized by Peptide Institute (Osaka, Japan) and used in the present study. ICV injection in the mudskipper brain has been established^21,44,49,50^. Anesthetized fish were injected post-orbitally along the midline into the third ventricle with 0.1 μl/g volume. The concentration of peptide solution was adjusted to 3 × 10^−8^ to 3 × 10^−5^ M. The doses were chosen based on the results of preliminary studies and published reports on effective physiological doses^21^. Fish treated with artificial cerebrospinal fluid served as handling controls. Evans blue (0.1%) was used to confirm the success of ICV injection. To minimize the leakage from the injection site, 30 sec was allowed to elapse after each injection. Fish were fully recovered from anesthetization in 1–2 min.

### Testing for amphibious behavior

Immediately after ICV injection with AngII alone (*n* = 7), ANP or BNP alone (*n* = 4–6), ANP in the presence of AngII (*n* = 4–7), or vehicle (*n* = 7), each fish was placed in the water area of experimental tank (Fig. 2a,b) as reported previously^21,44,50^. Water in the tank was constantly aerated, and plastic mesh on the land area facilitated drainage of water. The period in water and the frequency of migration between water and land area (defined as the ‘frequency of migration’) were recorded for 8 h.

### Drinking rate

As previously described^21^, fish were put in a columnar tank (diameter, 65 mm; height, 92 mm) after ICV injection with ANP (*n* = 5), BNP (*n* = 5), AngII (*n* = 6), ANP in the presence of AngII (*n* = 5–6), or vehicle (*n* = 6–8). The tank was filled with 0.004% phenol red in 10-ppt seawater so as not to expose fish to air. The amount of water in the whole gastrointestinal tract was measured according to the colorimetric method of previous studies^21,51^. Briefly, the whole tracts were dissected out and cut open into a petri dish. Phenol red was thoroughly washed from the tract by 1 ml saline. The 0.5-ml aliquots were mixed with 0.5 ml 5% trichloroacetic acid (Sigma-Aldrich), and centrifuged at 10,000 rpm for 5 min. The supernatant was mixed with 0.5 ml of 1 M NaOH, and absorbance was determined at 550 nm wave length by a spectrophotometer (DU640, Beckman Coulter, CA, USA).

### c-Fos immunohistochemistry

The brains were dissected out 1 h after ICV injection with 3 × 10^−6^ M AngII alone (*n* = 5), or 3 × 10^−5^ M ANP in the presence of AngII (*n* = 5). The procedure for the immunohistochemistry was previously described^21,52^. The brains were fixed in 4% paraformaldehyde in phosphate buffer (PB) and embedded in Paraplast (McCormick Scientific, Richmond, IL, USA). Serial sections were prepared at 7 μm and mounted onto MAS-coated slides (Matsunami Glass, Osaka, Japan). The sections were deparaffinized, rehydrated and then rinsed in phosphate buffered saline (PBS). The sections were immersed in methanol containing 0.3% H_2_O_2_ for 30 min at room temperature. The sections were rinsed thoroughly in PBS after the antigen activation and then pretreated with a blocking solution (2% normal goat serum, 0.01% NaN_3_ in PBS) for 1 h at room temperature and incubated with a polyclonal antibody raised against human c-Fos (1:200, sc-253, SantaCruz, Dallas, TX, USA) for 12 h at 4 °C. Specificity of the antibody against c-Fos protein of the mudskipper was previously confirmed by Western blotting analysis^52^. After rinsing in PBS, the sections were treated with ABC Elite kit (PK-6101, Vector, Burlingame, CA, USA) according to the manufacturer’s instruction. The sections were rinsed in 0.1 M PB and immersed in 0.01% 3,3′-diaminobenzidine tetrahydrochloride (DAB) in PB for 3 min to intensify colorization and then incubated in 0.01% DAB solution containing 0.01% H_2_O_2_ for 3 min in dark. Finally, the sections were rinsed in PB and distilled water, and immunoreactive c-Fos signals were examined and photographed using a digital camera (DMX1200; Nikon, Tokyo, Japan). The photomicrographs were binarized by graphic software Image J (https://imagej.nih.gov/ij/) and the number of c-Fos positive neurons was counted manually and compared between the cohorts. Every third section was analysed in order not to count positive cells repeatedly.

### Statistics

The data for amphibious behaviour, drinking rate, and the number of c-Fos immunoreactive cells are expressed as means ± standard error of the mean (s.e.m.). Kyplot 5.0 (KyensLab, Tokyo, Japan) was used for statistics analysis. The data for amphibious behaviour were analyzed with two-way or three-way repeated measures analysis of variance (ANOVA) followed by Tukey’s post-hoc test. The drinking rate was analysed with one-way factorial ANOVA followed by Dunnett’s post-hoc test or with Kruskal-Wallis followed by Steel-Dwass post-hoc test. Unpaired *t*-test was used for analyses of the number of c-Fos immunoreactive cells after the assumptions was checked.

## Supplementary information

Supplementary information

## Data Availability

Sequence data have been deposited into the DDBJ under accessions LC348996 for *anp* and LC348997 for *bnp*.
