# Supplementary material for: Hormonal regulation of thirst in the amphibious ray-finned fish suggests the requirement for terrestrialization during evolution
Source: Sci Rep. 2019 Nov 8;9:16347. doi: 10.1038/s41598-019-52870-7 (PMC6841719; doi:10.1038/s41598-019-52870-7)
Supplement: Supplementary file 1 — Supplementary information [file 41598_2019_52870_MOESM1_ESM.pdf]

Supplementary Information

Title: Hormonal regulation of thirst in the amphibious ray-finned fish suggests the requirement for terrestrialization during evolution

Author list and affiliations: Yukitoshi Katayama[1,2,*], Yoshio Takei[1], Makoto Kusakabe[1,3], Tatsuya Sakamoto[4]

[1]Laboratory of Physiology, Atmosphere and Ocean Research Institute, The University of Tokyo, 5-1-5 Kashiwanoha, Kashiwa, Chiba, 277-8564 Japan

[2]Faculty of Science, Toho University, 2-2-1 Miyama, Funabashi, Chiba, 274-8510 Japan

[3]Faculty of Science, Shizuoka University, 836 Otani, Suruga, Shizuoka, Shizuoka, 422-8529 Japan

[4]Ushimado Marine Institute, Faculty of Science, Okayama University, 130-17 Kashino, Setouchi, Okayama, 701-4303 Japan

[*]**Corresponding author:** Yukitoshi Katayama, Laboratory of Physiology, Atmosphere and Ocean Research Institute, University of Tokyo, 5-1-5 Kashiwanoha, Kashiwa, Chiba, 277-8564 Japan

E-mail address: katayama3g@aori.u-tokyo.ac.jp

Tel: +81-4-7136-6204, Fax: +81-4-7136-6206

Supplementary Methods

Expression of *anp* and *bnp* mRNAs during freshwater, seawater or terrestrial adaptation

As previously described [44, 50, 53-55], each intact mudskipper was transferred from 10-ppt seawater and placed into an individual aquarium containing freshwater (n = 8), 10-ppt seawater (control, n = 8), 30-ppt seawater (n = 7), or an aquarium without water (n = 8). Fishes in freshwater, 10-ppt seawater (control), or 30-ppt seawater conditions could climb on the land area of the aquarium *ad libitum*. At 5 h, based on studies in mudskippers indicating that many physiological changes occur at this time point following water loss of up to 20% of initial body mass [44, 50, 53-56], the hearts including the atriums and ventricles were immediately removed, frozen in liquid nitrogen, and then kept at −80 °C. The amount of *anp* and *bnp* mRNAs was determined by real-time quantitative PCR (qPCR) method using a 7900HT Sequence Detection System (Applied Biosystems, Carlsbad, CA, USA), as previously described in detail [57]. One µg of the extracted total RNA was treated with TURBO DNA-free (Ambion, Austin, TX, USA) and reverse-transcribed to first-strand cDNA using High Capacity cDNA Reverse Transcription kit. A 2 µl sample of cDNA template was added to 8 µl of reaction

mixture, and measurement was performed in duplicate. To generate a standard curve, plasmids containing partial cDNA fragments of target genes of known concentration were serially diluted and used as the standard templates. Total copy numbers of mRNAs in the heart were then calculated. Results for *anp* and *bnp* were normalized using the relatively constant *ef1α* mRNA levels. The following primers were designed using Primer Express software for *anp*, forward primer 5’-CGGATTGGGAACGCTAGT-3’ and reverse primer 5’-AAACAGCTGAAACAGGAGAAGA-3’ ; *bnp*, forward primer 5’-AATCCGTGAGCTCCTCTCTG-3’ and reverse primer 5’-GACTTGGGACTGTTGTTCCC-3’; and *ef1α*, forward primer 5’-ACATGCTCGAGACCAGTGAG-3’ and reverse primer 5’-CAGAGCCTCCAAGAGGGTAG-3’. The qPCR was performed with KAPA SYBR Fast qPCR kit (Kapa Biosystems, Boston, MA, USA). One-way repeated measures ANOVA was used for statistical analysis.

Supplementary Results

The number of c-Fos immunopositive neurons in the medial longitudinal fasciculus

(MLF)

We examined the effect of ANP and AngII on the c-Fos immunoreactivity at the MLF where the neurons were not involved in drinking [30]. There was no significant difference between injections in the number of c-Fos immunopositive neurons [AngII alone, 42.3 ± 15.3 (n = 5) vs. ANP with AngII, 35.1 ± 6.4 (n = 5)] with unpaired t-test.

Expression of *anp* and *bnp* mRNAs during environmental adaptation

There were no significant differences in the heart mRNA levels either of *anp* or *bnp* among terrestrial, seawater, and freshwater environments (Supplementary Table 1).

Supplementary Figures

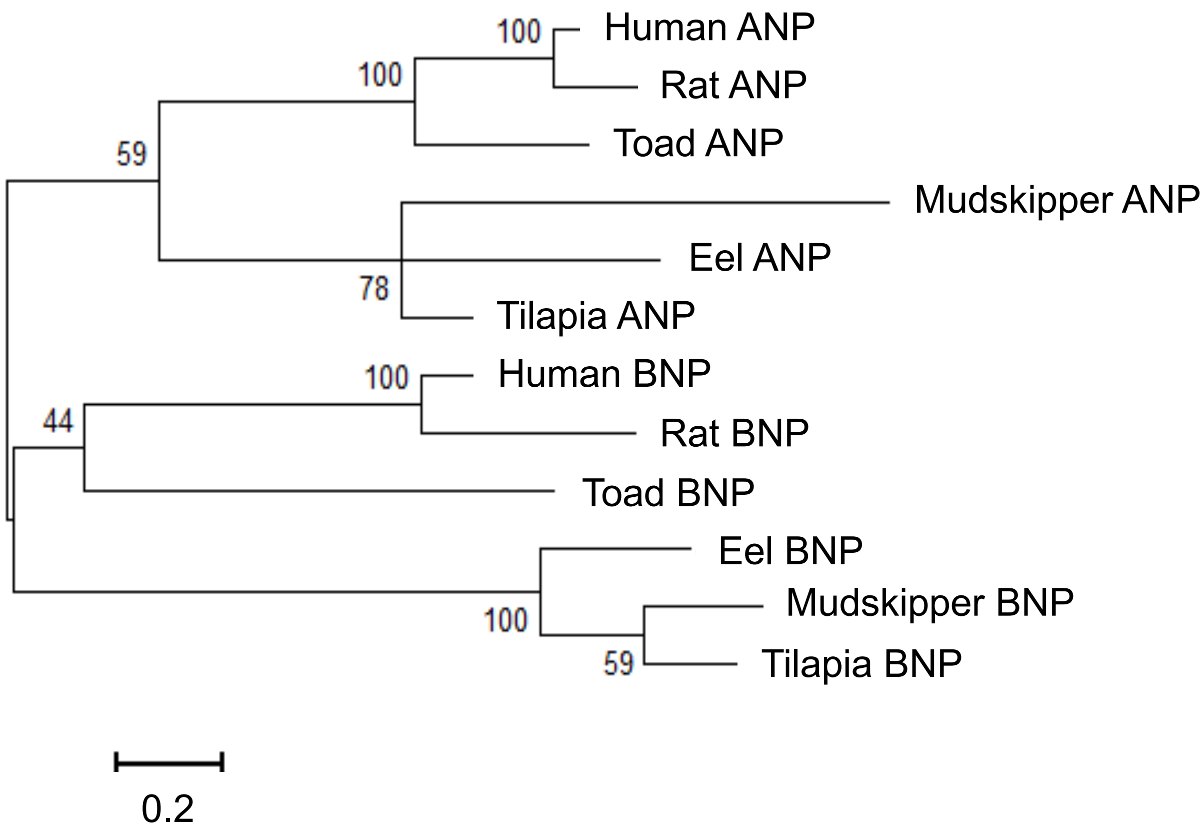

Supplementary Fig. 1 Molecular phylogenetic tree of natriuretic-peptide cDNA sequences.

Molecular phylogenetic tree was generated by maximum likelihood method. The numbers shown on the interior nodes are bootstrap values (percent).

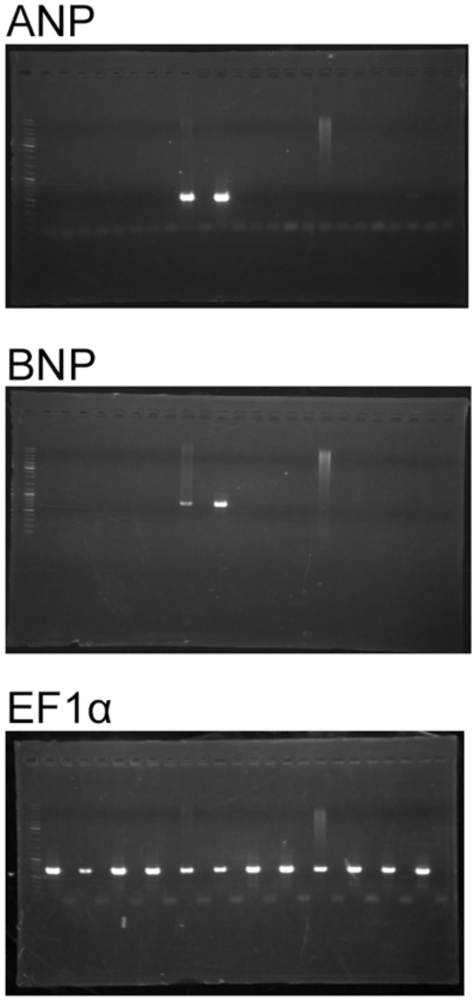

Supplementary Fig. 2 Full-length gels showing tissue distribution of *anp*, *bnp*, and *ef1α* mRNAs.

Supplementary Table

Supplementary Table 1 mRNA expression of *atrial natriuretic peptide* (*anp*) and *b-type natriuretic peptide* (*bnp*) in the heart of mudskippers under various environmental conditions and control (10 ppt seawater).

mRNA	Control	Terrestrial	Fresh water	30 ppt seawater
anp	11.4 ± 0.5	8.6 ± 1.1	10.2 ± 0.7	10.6 ± 1.2
bnp	1.1 ± 0.1	1.3 ± 0.2	1.0 ± 0.1	1.0 ± 0.3

Copy number of each mRNA is normalized using *ef1α*. There were no significant differences among environments in each of the mRNAs (*n* = 7-8 fishes). ppt: parts per thousand. Data are shown as means ± standard error of the mean.
